# Female Sex Workers in the Amazon Region of Brazil Are at High Risk of *Chlamydia trachomatis* Infection: A Retrospective Study

**DOI:** 10.3390/microorganisms13081815

**Published:** 2025-08-03

**Authors:** Leonardo Gabriel Campelo Pinto de Figueiredo, Paula do Socorro de Oliveira da Costa Laurindo, Daniela Assunção Pantoja, Maurimélia Mesquita da Costa, Diogo Oliveira de Araújo, Felipe Bonfim Freitas, Jacqueline Cortinhas Monteiro, Ricardo Roberto de Souza Fonseca, Rosimar Neris Martins Feitosa, Rogério Valois Laurentino, Leonardo Miranda dos Santos, Aldemir Branco Oliveira-Filho, Luiz Fernando Almeida Machado

**Affiliations:** 1 Biology of Infectious and Parasitic Agents Post-Graduate Program, Federal University of Pará, Belém 66075-110, PA, Brazil; liguerdo@gmail.com (L.G.C.P.d.F.); paula.biomedica@gmail.com (P.d.S.d.O.d.C.L.); danielaassuncao44@gmail.com (D.A.P.); lfam@ufpa.br (L.F.A.M.); 2 Virology Laboratory, Institute of Biological Sciences, Federal University of Pará, Belém 66075-110, PA, Brazil; diaraujo84@gmail.com (D.O.d.A.); jacqueline@ufpa.br (J.C.M.); ricardofonseca285@gmail.com (R.R.d.S.F.); rosimar@ufpa.br (R.N.M.F.); valois@ufpa.br (R.V.L.); 3 Evandro Chagas Institute, Health Ministry of Brazil, Ananindeua 67030-000, PA, Brazil; felipebonfim@iec.gov.br; 4 Molecular and Cellular Laboratory, Tropical Medicine Center, Federal University of Pará, Belém 66055-240, PA, Brazil; 5 Study and Research Group on Vulnerable Populations, Institute for Coastal Studies, Federal University of Pará, Bragança 68600-000, PA, Brazil; olivfilho@ufpa.br

**Keywords:** *Chlamydia trachomatis*, female sex workers, seroprevalence, Amazon, public health

## Abstract

Background: *Chlamydia trachomatis* is the most prevalent bacterial sexually transmitted infection (STI) globally, linked to severe complications such as pelvic inflammatory disease and infertility. In the Brazilian Amazon, socioeconomic vulnerability and the absence of screening policies exacerbate risks, particularly among female sex workers (FSWs). Objective: This study aimed to determine the seroprevalence of *anti-C. trachomatis* IgG antibodies among FSWs in five municipalities of Pará State, Brazilian Amazon, and identify epidemiological factors associated with infection. Methods: A retrospective cross-sectional study (2005–2007) included 348 FSWs recruited via convenience sampling. Sociodemographic and behavioral data were collected through questionnaires, and blood samples were analyzed by ELISA for *anti-C. trachomatis* IgG. Statistical analyses included Fisher’s exact tests, odds ratios (ORs), and 95% confidence intervals (CIs), using SPSS 21.0. Results: Overall seroprevalence was 93.9% (327/348; 95% CI: 83.1–90%). Significant associations included a household income of 1–3 minimum wages (98.4%; *p* = 0.0002), sexual partners from the same region (98.8%; *p* = 0.0421), and age >42 years (96.3%). Most reported inconsistent condom use (43.7%), multiple monthly partners (54.6%), and illicit drug use (53.4%). Discussion: The extremely high seroprevalence reflects chronic *C. trachomatis* exposure, driven by socioeconomic deprivation and limited healthcare access. Comparisons with global data underscore the urgent need for screening policies, absent in Brazil for FSWs, and highlight the vulnerability of this population. Conclusions: The findings reveal an alarming burden of *C. trachomatis* exposure among Amazonian FSWs. Integrated strategies, including routine screening, sexual health education, and inclusion of FSWs in priority health programs, are critical to reduce transmission and associated complications.

## 1. Introduction

*Chlamydia trachomatis* causes the most prevalent bacterial sexually transmitted infection (STI) in the world, with approximately 128.5 million new annual cases, predominantly affecting women aged 15–49 years [1]. It is classified into 19 genotypes that are involved in three different clinical conditions, such as trachoma, caused by genotypes A to C [2], lymphogranuloma venereum, caused by genotypes L1 to L3, and urogenital infection, caused by genotypes D to K [3], which can be asymptomatic in up to 80% of women and lead to pelvic inflammatory disease (PID), which is an ascending and systemic inflammatory/infectious process that manifests with salpingitis, endometritis, ovaritis, cervicitis, and urethritis [4,5]. Fibrosis caused by PID is responsible for tubal occlusion and other sequelae such as preventable infertility due to tubal factor and ectopic pregnancy [6]. In addition, it favors infection by the Human Immunodeficiency Virus (HIV) [7,8]. The highest rates of STIs caused by *C. trachomatis* are found in the United States (69.9–73.7%) [9,10], the Emirate of Abu Dhabi, and United Arab Emirates (18.9%) [11], followed by China (18.8%), Mexico (14.8%), Peru (13.9%), Germany (10.9%), Poland (7.4%) [12], and the Netherlands (10.5%) [13].

The Brazilian health system is universal and decentralized, and although there is an updated treatment protocol for STIs caused by *C. trachomatis* [14], it does not include asymptomatic young women of reproductive age, and the epidemiological scenario of *C. trachomatis* remains unnoticed, with hospitalization rates for PID exceeding 45,000 annually [15,16]. The Brazilian Amazon has low demographics and socioeconomic conditions, which impact the high socioeconomic vulnerability of hard-to-reach communities, and logistical and political–ideological problems hinder the adherence, territorialization, and expansion of primary health services in these locations [17,18]. The prevalence of anti-*C. trachomatis* antibodies in the Amazon has been reported as 33.3% (in STI clinics), 97.1% (in indigenous people of Parakanã village) [19], 30.9% in residents of Marajó Island [20], 64.8% in the population of people living with HIV [21], and 22.2% in individuals from a riverside community [22].

Female sex workers (FSWs) are a population with high vulnerability to STIs because they are often subjected to a lifestyle that exposes them to risk factors, such as multiple partners and group sex [23], discontinued condom use during sexual intercourse, and early sexual initiation [24]. In addition, asymptomatic FSWs seek sexual health services only when they have moderate or severe clinical symptoms [25]. There are few studies on STIs in FSWs in the Brazilian Amazon, but they report high rates of these infections in people in prostitution [26,27]. The investigation of anti-*C. trachomatis* serological markers in remote regions of the Amazon offers an important tool for assessing the exposure profile of women to sexual infection by *C. trachomatis*, as they are easy to perform and low-cost [28]. Therefore, the aim of this study was to identify IgG antibodies against *C. trachomatis* infection in FSWs from five municipalities in the state of Pará, the Amazon region of Brazil.

## 2. Material and Methods

### 2.1. Study Area, Design, and Ethics Aspects

**Figure 1:**
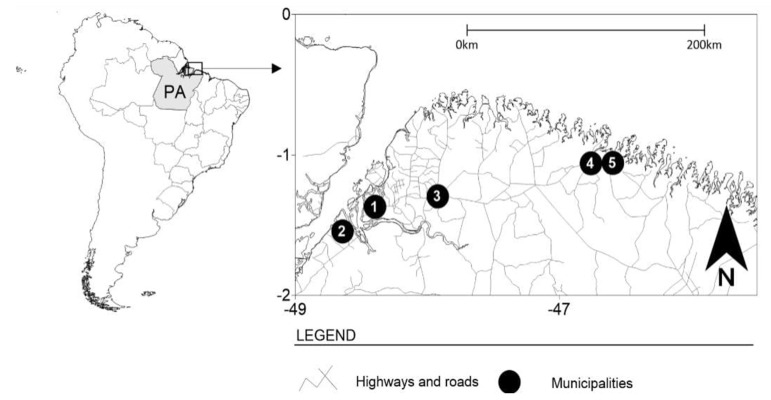
The municipalities where data collection was performed for this study. Belém—1, Barcarena—2, Castanhal—3, Bragança—4, and Augusto Corrêa—5.

This was a cross-sectional, retrospective, and analytical study, whose study population was FSWs from five locations in the state of Pará, northern Brazil: Belém (capital), Bragança, Augusto Corrêa, Barcarena, and Castanhal (Figure 1). These municipalities are areas of intense flow of people and circulation of products, with many historical, cultural, and tourist attractions that stand out in the context of the Amazon region; however, they are marked by areas of prostitution.

This study was approved by the Human Research Ethics Committee of the HEMOPA Foundation, under number 12/2005.

This study included FSWs and lasted from January 2005 to August 2007. Furthermore, a non-probabilistic convenience sample sampling method was used to recruit FSWs at their workplaces (streets, nightclubs, strip clubs, etc.) twice a week. To achieve an adequate sample size, we referred to previous prevalence in Belém do Pará (33%) [19]. The assumed sampling error (ε) was 5%, and the power of 80% was chosen, generating a minimum size of 345 participants. This study included cisgender women who engaged in sex work for money and had worked in the selected locations for at least six months. Transgender women, those under the influence of illicit drugs or alcohol at the time of data collection, and those unable to adequately respond to the epidemiological questionnaire were excluded. In each municipality included in this study, a data survey was carried out to identify and recognize the places that FSWs used as prostitution points. Subsequently, an invitation was made to the leaders of the FSW groups so that they could inform the other FSWs and extend the invitation to participate in this study. The variables investigated were as follows: age group (year), marital status, education level (years of study), family income (minimum wage), condom use, STI history, use of illicit drugs, number of partners (monthly), partners from another region, and partners from other countries.

The FSWs were informed of the objectives of this study prior to being invited to participate. The Informed Consent Form was signed by the participant after verbal agreement and acceptance. They provided information on social status and sexual behavior through the data collection questionnaire.

### 2.2. Laboratory Tests

A peripheral blood sample (5 mL) was collected from each FSW using a vacuum collection system with EDTA as an anticoagulant. The plasma was separated by centrifugation (9500 rpm for 15 min) and stored at −20 °C until use, in the Virology Laboratory of the Institute of Biological Sciences at the Federal University of Pará. Each sample was subjected to an enzyme-linked immunosorbent assay (ELISA) to detect specific IgG antibodies against *C. trachomatis* (Diagnostic Automation Inc.—Microwell Elisa, Calabasas, CA, USA; Specificity, 98.5%; Sensitivity, 91.1%) according to the manufacturer’s instructions. It is worth noting that all serological tests were performed at the time the samples were collected.

### 2.3. Statistical Analysis

Statistical analysis involved the correlation of serological results with the epidemiological data of the study population. The Statistical Package for Social Sciences (SPSS) version 21.0 (SPSS, Chicago, IL, USA) was used for analysis. The Proportion Parameter Estimation was used to verify the value of the proportion of positives over the total sample. For categorical variables with only two options, Fisher’s exact test and the odds ratio test were used. For variables with more than two options, the G test of independence was used. The 95% confidence interval (CI) was calculated for these comparisons, no adjustments for multiple comparisons (e.g., Bonferroni correction) were applied, as this study prioritized exploratory sensitivity over Type I error reduction in a high-prevalence context (93.9% seropositivity), where avoiding Type II errors (false negatives) was critical to identifying potential public health risks. A significance level of *p* ≤ 0.05 was considered for all analyses.

### 2.4. Ethics Statement

The investigations were conducted in accordance with the principles outlined in the Declaration of Helsinki (1975, revised in 2013), according to point 23 of this declaration and in accordance with Resolution 466/2012 of the Brazilian National Health Council [29]. This study is part of the project “Epidemiology of viral (HIV, HTLV, HBV and HCV) and bacterial (*T. pallidum* and *C. trachomatis*) infections in female sex workers in the states of Pará, Amapá and Acre, Northern region of Brazil”. The methodological steps of this study followed the pertinent ethical guidelines, as it was authorized by the Research Ethics Committee of the João de Barros Barreto University Hospital, Federal University of Pará (process number: 2089/05). In all cases, capital and interior, only participants who had read and signed the Free and Informed Consent Form (FICF) and were aged 18 years and older were included in this study. After this, data and biological sample collection continued while maintaining complete anonymity. Participants who tested positive for antibodies against *C. trachomatis* received appropriate guidance and referral for medical evaluation and treatment.

## 3. Results

A total of 348 FSWs participated in this study, with a mean age of 43.1 years (15 to 71 years), and most of the participants (61.2%; 213/348) were between 23 and 42 years of age. The total seroprevalence of antibodies against *C. trachomatis* infection was 93.9% (327/348; 95% CI: 83.1–90%), and in relative frequency, it was found significantly in women who had a family income between one and three Brazilian minimum wages (98.4%, *p* = 0.0002) and had fewer than 20 sexual partners per month (94.3%), with partners from the same region (98.8%, *p* = 0.0421) and from this country (95.5%). The epidemiological information of the FSWs participating in this study is contained in Table 1.

## 4. Discussion

In this study, we found high seroprevalence of antibodies against infection by *C. trachomatis* in FSWs from the capital and four other municipalities of the state of Pará, in the Amazon region of Brazil. The seroprevalence observed here far exceeds rates reported in asymptomatic populations from other countries [11,12,13,28,30], most of which are from developed countries that have an official screening policy for *C. trachomatis* in young adult women [31,32,33]. Interestingly, high seroprevalences were found in American women with active clinical disease with evident symptoms of PID (73.7%) [9] and tubal factor infertility (69.9%) [10]. We did not assess the clinical conditions or gynecological complaints of the participants to verify any correlation. High seroprevalence rates were also found in female populations with socioeconomic vulnerability, such as HIV-positive married women in Nigeria (60.9%) [34], and asymptomatic women without access to health services in Puerto Rico (47%) [35] and China (47.46%) [36].

Most FSWs are single mothers and therefore choose a larger number of clients to ensure higher income, guarantee the subsistence of their families and fit within the average Brazilian family income, which is approximately BRL 2927.70, equivalent to USD 526.70 [37]. This may be reflected in our study in the high levels of antibodies against *C. trachomatis* found in women who had a family income between one and three Brazilian minimum wages (equivalent to USD 250–USD 750) (*p* = 0.0002), possibly due to frequent exposure to this infection. In Brazil, socioeconomic instability associated with high STI rates is strongly present in peripheral societies and is commonly reported as a factor that favors risky sexual behavior [38,39] and encourages the activity of exchanging sex for money or goods [40,41,42].

The populations of the Amazon suffer from basic deficiencies in prevention, promotion, diagnosis, and health education and are constantly reported with high prevalence of this and other STIs [16,17]. Furthermore, we consider that the understanding of the epidemiology of *C. trachomatis* in Brazil is the “tip of the iceberg”, as it is based only on studies in isolated regions. Since its first notification in 1988 [43], STI caused by *C. trachomatis* in the Amazon has been characterized by high seroprevalence among symptomatic patients, in patients recently diagnosed with HIV (64.8%) [21], and in indigenous villages (23.9–90.7%) [44]. High frequencies continue to be confirmed today with nucleic acid amplification tests in the Marajó region (4%) [45], in gynecological clinic attendees (4.6%) [46], public maternity hospitals (11–18%) [47,48], and in asymptomatic university students (11.2%) [49].

Since 1984, Brazil has been concerned with including the promotion of women’s reproductive health in primary care [50], but there are no public policies aimed at FSW reproductive health in the National Program for Comprehensive Women’s Health Care (PNAISM) even after the universalization of the health system [51,52], and their participation in debates for the construction and/or reformulation of health policies is still rare. In addition, the logistical difficulties and political–administrative problems suffered by populations in remote and hard-to-reach regions of the Amazon are an aggravating factor to be considered [17,18]. In Brazil, FSWs work as sex workers in their own businesses and in small groups [53], but a systematic commercial network with mediators, promoters, or assistants of sex work, such as the creation of “brothels”, procuring and pimping websites, is a crime [54]. The vast majority of them use the streets, highways, and rivers of the Amazon, which have a large flow of people, as temporary professional addresses, since travelers or men from other locations in the same region are routinely associated with the use of FSWs’ services [26,27]. Perhaps for this reason, our study showed that FSWs who had partners from this region had a higher seroprevalence of *C. trachomatis* (*p* = 0.0421), and many of them have little access to and reception in health services or do not declare themselves to be FSWs [54,55], due to stigmatization and social discrimination [56], which leads to hesitation in seeking these services for this population group [57,58].

The main limitations of this study were the limited sample size with the use of secondary data and the occurrence of incomplete or lost medical records that prevented us from performing multivariate analyses. Another limitation in our study is the lack of clinical correlation with *C. trachomatis* seropositivity. Future clinical studies are needed to verify the natural history of this infection to adverse clinical outcomes such as PID, infertility, and HIV co-infection. In addition, there is a possibility of cross-reaction in women who had previous trachoma events and who had a false-positive result, as well as in participants who were affected by active asymptomatic sexual infection at the time of this study, which may have implications for the false-negative result.

## 5. Conclusions

We concluded that FSWs who had a family income of one to three Brazilian minimum wages and those who receive clients from other states showed higher seroprevalence for anti-*C. trachomatis* antibodies. Brazilian public health policies should implement routine *C. trachomatis* screening programs for women and include FSWs as a priority group in official women’s health programs, which should be complemented by innovative actions for sexual and reproductive health education. These actions should be intensified in the FSW population, as this is a population group that is strongly present in Brazilian society and deserves to be integrated into STI prevention measures. Future studies will be necessary to verify a possible association between associated tubal immunopathologies and high seropositivity for antibodies against *C. trachomatis* infection in FSWs.

## Figures and Tables

**Table 1:** Seroprevalence, epidemiological characteristics, and sexual behavior of female sex workers included in this study.

Characteristics	Total (n = 348)		CT IgG+ (n = 327)		*p*-Value	Odds Ratio	(95% CI)	*p*-Value
	N	%	N	%				
Age groups (years) ^a^					0.7063	-	-	-
15–22	108	31.0	100	92.6				
23–42	213	61.2	201	94.4				
>42	27	7.8	26	96.3				
Marital status ^a^					0.0820	-	-	-
Single	277	79.6	264	95.3				
Married	56	16.1	51	91				
Widowed/divorced	15	4.3	12	80				
Education level (years of study) ^b^					0.8058	1.2152	0.4325–3.4144	0.9064
<8	253	72.7	237	93.7				
>8	95	27.3	90	95.7				
Family income (minimum wage) ^a^					0.0002	-	-	-
<1	121	34.8	105	86.8				
1–3	185	53.2	182	98.4				
>3	42	12.0	40	95.2				
Condom use ^b^					0.5845	0.9982	0.4093–2.4347	0.8231
Yes	196	56.3	187	95.4				
No	152	43.7	140	92.1				
STI history ^b^					0.8480	1.1068	0.3921–3.1241	0.9367
Yes	77	22.1	72	93.5				
No	271	77.9	255	94				
Use of illicit drugs ^b^					0.9009	0.9554	0.3949–2.3116	0.9009
Yes	186	53.4	175	94				
No	162	46.6	152	93.8				
Nº. of partners (monthly) ^b^					0.8090	0.8960	0.3675–2.1846	0.9876
<20	158	45.4	149	94.3				
≥20	190	54.6	178	93.6				
Partners from another region ^a^					0.0421	-	-	-
Yes	204	58.6	188	92.1				
No	85	24.4	84	98.8				
Not known	59	17.0	55	93.2				
Partners from other countries ^a^					0.3661	-	-	-
Yes	119	34.2	112	94.1				
No	155	44.5	148	95.5				
Not known	74	21.3	67	90.5				

^a^ G of Independence test, ^b^ Fisher’s exact. The 95% confidence interval (CI) was used for these analyses, and a *p* ≤ 0.05 significance level was considered.

## Data Availability

The original contributions presented in this study are included in the article. Further inquiries can be directed to the corresponding author.

## References

[1] Li C., Ong J., Tang W., Wang C. (2023). *Chlamydia trachomatis* infection: Epidemiology, prevention, clinical, and basic science research. Front. Public Health.

[2] Tedijanto C., Solomon A.W., Martin D.L., Nash S.D., Keenan J.D., Lietman T.M., Lammie P.J., Aiemjoy K., Amza A., Aragie S. (2023). Monitoring transmission intensity of trachoma with serology. Nat. Commun..

[3] Grygiel-Górniak B., Folga B.A. (2023). *Chlamydia trachomatis*—An Emerging Old Entity?. Microorganisms.

[4] Heijer C.D.J., Hoebe C.J.P.A., Driessen J.H.M., Wolffs P., Broek I.V.F., Hoenderboom B.M., Williams R., Vries R., Dukers-Muijrers N.H.T.M. (2019). *Chlamydia trachomatis* and the Risk of Pelvic Inflammatory Disease, Ectopic Pregnancy, and Female Infertility: A Retrospective Cohort Study Among Primary Care Patients. Clin. Infect. Dis..

[5] Hunt S., Vollenhoven B. (2023). Pelvic inflammatory disease and infertility. Aust. J. Gen. Pract..

[6] Tang W., Mao J., Li K.T., Walker J.S., Chou R., Fu R., Chen W., Darville T., Klausner J., Tucker J.D. (2020). Pregnancy and fertility-related adverse outcomes associated with *Chlamydia trachomatis* infection: A global systematic review and meta-analysis. Sex. Transm. Infect..

[7] Veretennikova A., Chang T.L. (2022). *Chlamydia trachomatis* Enhances HIV Infection of Non-Activated PBMCs. EC Microbiol..

[8] Dzakah E.E., Zhao J., Wang L., Rashid F., Xu R., Yang L., Wan Z., Huang L., Wang H., Chen S. (2022). *Chlamydia trachomatis* Stimulation Enhances HIV-1 Susceptibility through the Modulation of a Member of the Macrophage Inflammatory Proteins. J. Investig. Dermatol..

[9] Anyalechi G.E., Hong J., Danavall D.C., Martin D.L., Gwyn S.E., Horner P.J., Raphael B.H., Kirkcaldy R.D., Kersh E.N., Bernstein K.T. (2021). High Plasmid Gene Protein 3 (Pgp3) *Chlamydia trachomatis* Seropositivity, Pelvic Inflammatory Disease, and Infertility Among Women, National Health and Nutrition Examination Survey, United States, 2013–2016. Clin. Infect. Dis..

[10] Anyalechi G.E., Hong J., Kirkcaldy R.D., Wiesenfeld H.C., Horner P., Wills G.S., McClure M.O., Hammond K.R., Haggerty C.L., Kissin D.M. (2022). Chlamydial Pgp3 Seropositivity and Population-Attributable Fraction Among Women with Tubal Factor Infertility. Sex. Transm. Dis..

[11] Abdo N.M., Aslam I., Irfan S., George J.A., Alsuwaidi A.R., Ahmed L.A., Al-Rifai R.H. (2023). *Chlamydia trachomatis* Seroepidemiology and Associated Factors in Fertility Treatment-Seeking Patients in the Abu Dhabi Emirate, United Arab Emirates. Sex. Transm. Dis..

[12] Warnecke J.M., Pollmann M., Borchardt-Lohölter V., Moreira-Soto A., Kaya S., Sener A.G., Gómez-Guzmán E., Figueroa-Hernández L., Li W., Li F. (2020). Seroprevalences of antibodies against ToRCH infectious pathogens in women of childbearing age residing in Brazil, Mexico, Germany, Poland, Turkey and China. Epidemiol. Infect..

[13] Alexiou Z.W., van Aar F., Hoenderboom B.M., Morre S.A., Heijne J.C.M. (2024). Trends in *Chlamydia trachomatis* IgG seroprevalence in the general population of the Netherlands over 20 years. Sex. Transm. Infect..

[14] Brasil Ministério da Saúde, Secretaria de Vigilância em Saúde, Departamento de Doenças de Condições Crônicas e Infecções Sexualmente Transmissíveis (2022). Protocolo Clínico e Diretrizes Terapêuticas para Atenção Integral às Pessoas com Infecções Sexualmente Transmissíveis—IST [Recurso Eletrônico].

[15] Menezes M.L.B., Giraldo P.C., Linhares I.M., Boldrini N.A.T., Aragón M.G. (2021). Brazilian Protocol for Sexually Transmitted Infections 2020: Pelvic inflammatory disease. Epidemiol. Serv. Saude.

[16] Perciney P., Costa A.L.S., Leite I.C.G., Nogueira M.C. (2022). Pelvic inflammatory disease hospitalizations in Brazil: Time trend from 2000 to 2019. Rev. Bras. Saude Mater. Infant..

[17] Machado L.F.A., Fonseca R.R.S., Oliveira-Filho M.A.F., Cayres-Vallinoto I.M.V., Vallinoto A.C.R., de Oliveira Guimarães Ishak M., Ishak R. (2021). The Epidemiological Impact of STIs among General and Vulnerable Populations of the Amazon Region of Brazil: 30 Years of Surveillance. Viruses.

[18] Faria R.M. (2020). The territorialization of Primary Health Care of the Brazilian Unified Health System. Cien. Saude Colet..

[19] Ishak M.O., Ishak R., Cruz A.C., Santos D.E., Salgado U. (1993). Chlamydial infection in the Amazon region of Brazil. Trans. R. Soc. Trop. Med. Hyg..

[20] Ferreira G.R.O.N., Freitas F.B., Queiroz M.A.F., Lima S.S., Vallinoto A.C.R., de O Guimarães Ishak M., Ishak R. (2019). Epidemiology and risk factors for *Chlamydia trachomatis*, treponema pallidum, Hepatitis B Virus and Hepatitis C Virus in the Marajó Archipelago, Brazilian Amazon. J. Community Med. Health Educ..

[21] Góes S.D.S., Fonseca R.R.S., Avelino M.E.S., Lima S.S., Lima M.S.G.A., Laurentino R.V., Queiroz M.A.F., Freitas F.B., Vallinoto A.C.R., Ishak R. (2022). Exposure to *Chlamydia trachomatis* Infection in Individuals Who Are Newly Diagnosed with HIV and Antiretroviral-Naive from Belem, Northern Brazil. Vaccines.

[22] Galvão J.J.D.S., Cunha C.L.F., Pinho E.C.C., Paiva D.J.D.S., de Castro N.J.C., Nascimento V.G.C., de Azevedo Junior W.S., da Silva R.A.R., Feitosa R.N.M., Vallinoto A.C.R. (2022). Seroprevalence of *Chlamydia trachomatis* and Associated Factors among Vulnerable Riverine in the Brazilian Amazon. Int. J. Environ. Res. Public Health.

[23] Shi L., Luo J., Chen Y., Chen L., Hu H., Qiu T., Liu X., Xu X., Chen Y., Zhang Z. (2022). Prevalence of syphilis and *Chlamydia trachomatis* infection among female sex workers in Jiangsu, China: Results from a multicenter cross-sectional and venue-based study. Front. Public Health.

[24] Tremblay F., Courtemanche Y., Bélanger R.E., Turcotte-Tremblay A.M. (2024). A systematic review of the association between history of sexually transmitted infections and subsequent condom use in adolescents. BMC Public Health.

[25] Birger L., Peled E., Benyamini Y. (2024). Stigmatizing and inaccessible: The perspectives of female sex workers on barriers to reproductive healthcare utilization—A scoping review. J. Adv. Nurs..

[26] Oliveira-Filho A.B., Aires D.W.F., Cavalcante N.S., Raiol N.C., Lisboa B.L.A., Frade P.C.R., da Costa L.M., Pinheiro L.M.L., Machado L.F.A., Martins L.C. (2019). Hepatitis C Virus among Female Sex Workers: A Cross-Sectional Study Conducted along Rivers and Highways in the Amazon Region. Pathogens.

[27] Coelho E.C., Souza S.B., Costa C.C.S., Costa L.M., Pinheiro L.M.L., Machado L.F.A., Silva-Oliveira G.C., Martins L.C., Frade P.C.R., Oliveira-Filho A.B. (2021). Treponema pallidum in female sex workers from the Brazilian Marajo Archipelago: Prevalence, risk factors, drug-resistant mutations and coinfections. Trans. R. Soc. Trop. Med. Hyg..

[28] Petersen M.R., Patel E.U., Grabowski M.K., Gaydos C.A., Quinn T.C., Tobian A.A.R. (2021). Seroprevalence of *Chlamydia trachomatis* Among Female Adults in the United States: The National Health and Nutrition Examination Surveys. Clin. Infect. Dis..

[29] Conselho Nacional de Saúde (Brasil) Resolução N° 466, de 12 de Dezembro de 2012. Brasília, 2012. [citado 11 March 2014]. https://www.gov.br/conselho-nacional-de-saude/pt-br.

[30] Öhman H., Rantsi T., Joki-Korpela P., Tiitinen A., Surcel H.M. (2020). Prevalence and persistence of *Chlamydia trachomatis*-specific antibodies after occasional and recurrent infections. Sex. Transm. Infect..

[31] U.S. Preventive Services Task Force (2007). Screening for Chlamydial Infection: U.S. Preventive Services Task Force Recommendation Statement. Ann. Intern. Med..

[32] European Centre for Disease Prevention and Control (2014). Chlamydia Control in Europe: Literature Review.

[33] Low N., Hocking J.S., van Bergen J. (2021). The changing landscape of chlamydia control strategies. Lancet.

[34] Omosigho P.O., Ajide T.E., Izevbuwa O.E., Okesanya O.J., Oladejo J.M., Uyigue P.O. (2024). Seroprevalence of *Chlamydia trachomatis* and associated risk factors among HIV positive women in North Central Nigeria. Infez. Med..

[35] Castañeda-Avila M.A., Suárez-Pérez E., Bernabe-Dones R., Unger E.R., Panicker G., Ortiz A.P. (2020). *Chlamydia trachomatis* and Human Papillomavirus Serostatus in Puerto Rican Women. P. R. Health Sci. J..

[36] Zhou Q., Li J., Luo L., Min S., Wang L., Peng L., Hou Y., He P., He S., Tang S. (2024). Characterization of genital *Chlamydia trachomatis* infection among women attending infertility and gynecology clinics in Hunan, China. BMC Infect. Dis..

[37] Brasil Instituto de Pesquisa Econômica Aplicada Renda Média do Trabalhador Brasileiro Cresce 4.0% no Primeiro Trimestre de 2024 na Comparação com o Primeiro Trimestre de 2023. https://www.ipea.gov.br/portal/categorias/45-todas-as-noticias/noticias/15092-renda-media-do-trabalhador-brasileiro-cresce-4-0-no-primeiro-trimestre-de-2024-na-comparacao-com-o-primeiro-trimestre-de-2023.

[38] Tabler J., Mykyta L., Schmitz R.M., Kamimura A., Martinez D.A., Martinez R.D., Flores P., Gonzalez K., Marquez A., Marroquin G. (2019). Social Determinants of Sexual Behavior and Awareness of Sexually Transmitted Infections (STI) Among Low-Income HIV+ or STI At-Risk Hispanic Residents Receiving Care at the U.S.-Mexico Border. J. Community Health.

[39] Jiménez-Morón A., Hueso-Montoro C., Caparros-González R., Pérez-Morente M.Á. (2024). Risk factors for the acquisition of Sexually Transmitted Infections in sex workers: A systematic review. Rev. Esp. Salud Publica.

[40] Karandikar S., Gezinski L.B., Meshelemiah J.C. (2013). A qualitative examination of women involved in prostitution in Mumbai, India: The role of family and acquaintances. Int. Soc. Work..

[41] Footer K.H.A., White R.H., Park J.N., Decker M.R., Lutnick A., Sherman S.G. (2020). Entry to Sex Trade and Long-Term Vulnerabilities of Female Sex Workers Who Enter the Sex Trade Before the Age of Eighteen. J. Urban Health.

[42] Yoosefi Lebni J., Irandoost S.F., Dehghan A.A., Ziapour A., Khosravi B., Mehedi N. (2021). Exploring the reasons for women to engage in sex work in Tehran, Iran: A qualitative study. Heliyon.

[43] Ishak MO G., Mumtaz G., Ishak R., Ridgway G.L. (1988). Prevalence of antibodies to *Chlamydia trachomatis* in population groups of Brazil, England and Portugal. Rev. Inst. Med. Trop. Săo Paulo.

[44] Ishak Mde O., Costa M.M., Almeida N.C., Santiago A.M., Brito W.B., Vallinoto A.C., Azevedo V.N., Ishak R. (2015). *Chlamydia trachomatis* serotype A infections in the Amazon region of Brazil: Prevalence, entry and dissemination. Rev. Soc. Bras. Med. Trop..

[45] Santos L.M., Vieira M.R.M.D.S., Oliveira J.F.G., Trindade J.Q., Brasiliense D.M., Ferrari S.F., Tsutsumi M.Y., Fuzii H.T., Junior E.C.S., Ishikawa E.A.Y. (2018). High prevalence of sexual *Chlamydia trachomatis* infection in young women from Marajó Island, in the Brazilian Amazon. PLoS ONE.

[46] Santos L.M., Souza J.D., Mbakwa H.A., Nobre A.F.S., Vieira R.C., Ferrari S.F., Rodrigues A.R., Ishikawa E.A.Y., Guerreiro J.F., Sousa M.S. (2022). High prevalence of sexual infection by human papillomavirus and *Chlamydia trachomatis* in sexually-active women from a large city in the Amazon region of Brazil. PLoS ONE.

[47] Santos L.M., Souza I.R.A., Holanda L.H.C., Vaz J.O., Tsutsumi M.Y., Ishikawa E.A.Y., de Sousa M.S. (2016). Alta incidência da infecção urogenital por *Chlamydia trachomatis* em mulheres parturientes de Belém, Estado do Pará, Brasil. Rev. Pan-Amaz. Saúde.

[48] Brasiliense D.M., Borges B.N., Ferreira W.A. (2016). Genotyping and prevalence of *Chlamydia trachomatis* infection among women in Belém, Pará, northern Brazil. J. Infect. Dev. Ctries.

[49] Santos L.M.S., Vieira M.R.M.S., Vieira R.C., Silva L.B.L., Macêdo G.M.M., Miranda A.E., Brasiliense D.M., Guimarães R.J.P.S., Sousa Junior E.C., Ferrari S.F. (2024). Prevalence and circulant genotypes of *Chlamydia trachomatis* in university women from cities in the Brazilian Amazon. PLoS ONE.

[50] Brasil Ministério da Saúde (1984). Assistência Integral à Saúde da Mulher: Bases da Ação Programática.

[51] Brasil. Lei N° 8.080 de, de 19 de Setembro de 1990. Dispõe Sobre as Condições para a Promoção, Proteção e Recuperação da Saúde, a Organização e o Funcionamento dos Serviços Correspondentes e dá Outras Providências. http://www.planalto.gov.br/ccivil_03/leis/L8080.htm.

[52] Brasil Ministério da Saúde (2004). Secretaria de Atenção à Saúde. Departamento de Ações Programáticas Estratégicas. Política Nacional de Atenção Integral à Saúde da Mulher: Princípios e Diretrizes.

[53] Brasil Ministério do Trabalho—MST Código Brasileiro de Ocupações N° 5198-05 (Profissionais do Sexo) de 02 de Fevereiro de 2015. https://cbo.mte.gov.br/cbosite/pages/home.jsf.

[54] BRASIL. Decreto-Lei 2.848, de 07 de Dezembro de 1940. Código Penal. https://www2.camara.leg.br/legin/fed/declei/1940-1949/decreto-lei-2848-7-dezembro-1940-412868-publicacaooriginal-1-pe.html.

[55] Dourado I., Guimarães M.D.C., Damacena G.N., Magno L., Souza Júnior P.R.B., Szwarcwald C.L. (2019). Sex work stigma and non-disclosure to health care providers: Data from a large RDS study among FSW in Brazil. BMC Int. Health Hum. Rights.

[56] Pastori B.G., Colmanetti A.B., Aguiar C.A. (2022). Perceptions of sex workers about the care received in the health care context. J. Hum. Growth Dev..

[57] Lima F.S., Merchán-Hamann E., Urdaneta M., Damacena G.N., Szwarcwald C.L. (2017). Factors associated with violence against female sex workers in ten Brazilian cities. Cad. Saude Publica.

[58] Muhindo R., Mujugira A., Castelnuovo B., Sewankambo N.K., Parkes-Ratanshi R., Tumwesigye N.M., Nakku-Joloba E., Kiguli J. (2021). I felt very small and embarrassed by the health care provider when I requested to be tested for syphilis”: Barriers and facilitators of regular syphilis and HIV testing among female sex workers in Uganda. BMC Public Health.

